# Pathophysiological Link and Treatment Implication of Heart Failure and Preserved Ejection Fraction in Patients with Chronic Kidney Disease

**DOI:** 10.3390/biomedicines12050981

**Published:** 2024-04-30

**Authors:** Giacomo Bonacchi, Valentina Alice Rossi, Manuel Garofalo, Rocco Mollace, Giuseppe Uccello, Paolo Pieragnoli, Luca Checchi, Laura Perrotta, Luca Voltolini, Giuseppe Ricciardi, Matteo Beltrami

**Affiliations:** 1 Cardiomyopathy Unit, Careggi University Hospital, 50134 Florence, Italy; giacomobonacchi1@gmail.com; 2 Department of Cardiology, University Hospital of Zurich, 8091 Zurich, Switzerland; valereds@gmail.com; 3 Arrhythmia and Electrophysiology Unit, Careggi University Hospital, 50134 Florence, Italy; maanu.507@gmail.com (M.G.); pieragnolip@aou-careggi.toscana.it (P.P.); checchi.luca@gmail.com (L.C.); perrottal@aou-careggi.toscana.it (L.P.); ricciardig@aou-careggi.toscana.it (G.R.); 4 Department of Experimental Medicine, Tor Vergata University, 00133 Rome, Italy; rocco.mollace@gmail.com; 5 Cardiology Unit, Humanitas Gavazzeni, 24125 Bergamo, Italy; 6 Division of Cardiology, “A. Manzoni” Hospital—ASST Lecco, 23900 Lecco, Italy; giuseppe.uccello@yahoo.it; 7 Department of Experimental and Clinical Medicine, University of Florence, 50134 Florence, Italy; luca.voltolini@unifi.it; 8 Thoracic Surgery Unit, Careggi University Hospital, 50134 Florence, Italy

**Keywords:** heart failure preserved ejection fraction, chronic kidney disease, hyperkalemia, sodium–glucose-linked transporters 2 inhibitors, treatment, angiotensin receptor blocker, neprilysin inhibitors

## Abstract

Heart failure with preserved ejection fraction (HFpEF) results from a complex interplay of age, genetic, cardiac remodeling, and concomitant comorbidities including hypertension, obesity, diabetes, and chronic kidney disease (CKD). Renal failure is an important comorbidity of HFpEF, as well as a major pathophysiological mechanism for those patients at risk of developing HFpEF. Heart failure (HF) and CKD are intertwined conditions sharing common disease pathways; the so-called “kidney tamponade”, explained by an increase in intracapsular pressure caused by fluid retention, is only the latest model to explain renal injury in HF. Recognizing the different phenotypes of HFpEF remains a real challenge; the pathophysiological mechanisms of renal dysfunction may differ across the HF spectrum, as well as the prognostic role. A better understanding of the role of cardiorenal interactions in patients with HF in terms of symptom status, disease progression, and prognosis remains essential in HF management. Historically, patients with HF and CKD have been scarcely represented in clinical trial populations. Current concerns affect the practical approach to HF treatment, and, in this context, physicians are frequently hesitant to prescribe and titrate both new and old treatments. Therefore, the extensive application of HF drugs in diverse HF subtypes with numerous comorbidities and different renal dysfunction etiologies remains a controversial matter of discussion. Numerous recently introduced drugs, such as sodium–glucose-linked transporter 2 inhibitors (SGLT2i), constitute a new therapeutic option for patients with HF and CKD. Because of their protective vascular and hormonal actions, the use of these agents may be safely extended to patients with renal dysfunction in the long term. The present review delves into the phenotype of patients with HFpEF and CKD from a pathophysiological perspective, proposing a treatment approach that suggests a practical stepwise algorithm for the proper application of life-saving therapies in clinical practice.

## 1. Introduction

Heart failure with preserved ejection fraction (HFpEF) identifies a syndrome characterized by specific signs and symptoms due to myocardial dysfunction, which results in an increase in left-sided filling pressures despite a preserved left ventricular ejection fraction (LVEF) higher than 50% [1]. The traditional classification based on LVEF has clear therapeutic implications, considering the wide spectrum of drugs that have been proven effective for heart failure with reduced ejection fraction (HFrEF). On the other hand, there are few therapeutic options for HFpEF, and the inefficacy of many drug classes in HFpEF can be ascribed to its related variety of clinical scenarios and etiologies [2]. This is especially true for the so-called secondary HFpEF, or HFpEF mimics, namely, because of an identifiable condition (e.g., infiltrative cardiac disease, hypertrophic cardiomyopathy), that benefits from specific treatments [3]. However, it is equally important to characterize patients who develop HFpEF related to a combination of different causal, comorbidity-driven factors leading to a primary impairment in myocardial relaxation or compliance [4]. Guidelines stress the importance of identifying risk factors, specific etiologies, and comorbidities, suggesting that the treatment of the underlying phenotypes leads to improved outcomes in HFpEF [1]. In this context, chronic kidney disease (CKD) and heart failure (HF) are frequent conditions, both bearing a shared burden of conventional cardiovascular risk factors, such as hypertension and diabetes [5]. Common HF treatments such as renin–angiotensin system (RAAS) inhibitors, mineralcorticoid receptor antagonists (MRAs), angiotensin receptor blocker neprilysin inhibitors (ARNI), and SGLT2i significantly impact renal physiology. Indeed, these drugs modify the renal function curve, affecting the intraglomerular hydrostatic pressure–natriuresis relationship through the tubule–glomerular feedback mechanism as well as by inhibiting RAAS effects on the afferent and efferent glomerular arteriola. As a consequence, the physiological filtration fraction is directly influenced, determining a repercussion in baroceptorial and chemotactic control directed by the macula densa and affecting tubular function. The use of SGLT2i, diuretics, and concomitant administration of RAAS inhibitors and MRAs in specific phenotypes of HFpEF exacerbates the transient renal impairment observed after their initial administration. This phenomenon contributes to the reluctance to initiate and titrate these medications.

In this review, we discuss the pathophysiological link between HFpEF and CKD and the effects of HF drugs on the kidney, suggesting a practical stepwise algorithm for the correct application of these lifesaving therapies in clinical practice.

## 2. The Importance of Patient Profiling in HFpEF

The recent Heart Failure Association–European Heart Rhythm Association of the European Society of Cardiology–European Society of Hypertension joint scientific statement identifies the most common primary HFpEF phenotypes and proposes an algorithm to profile patients and tailor evidence-based treatments [3]. Several factors are intertwined and contribute to the development of HFpEF: from age and gender to disorders of blood pressure, heart rate (HR), and rhythm or weight to specific comorbidities like diabetes mellitus, chronic obstructive pulmonary disease (COPD), coronary artery disease (CAD), and CKD. Among these, age is one of the most significant factors, and defines two “true” HFpEF phenotypes [6] as follows: one is typically represented by younger patients with obesity and diabetes, more frequently males, with a predominance of Black and Asian races and a lower comorbidity burden but a worse lifestyle with respect to cardiovascular risk factors, and, thus, more likely to die from cardiovascular causes. The other age-related HFpEF phenotype is usually characterized by older patients, mainly white women, with a higher comorbidity burden, mostly represented by arterial hypertension, atrial fibrillation (AF), CKD, and frailty, who are more likely to die from non-cardiovascular death. Sex–gender is also an important element, considering that, women usually present with specific anatomic features (e.g., small ventricular chambers) [7]. Furthermore, women with HFpEF usually develop more severe symptoms and a poorer quality of life as compared with men [8].

Arterial hypertension is the most frequent cardiovascular risk factor associated with HFpEF. Similar pathophysiological mechanisms underlie both HFpEF and CKD, such as over-activation of the renin–angiotensin–aldosterone and sympathetic nervous systems and increased oxidative stress, which favor the onset of arterial stiffness and can induce left ventricular (LV) hypertrophy, myocardial fibrosis, and diastolic dysfunction [9,10]. The presence of AF is highly predictive for HFpEF and must be considered in the pretest assessment of the HFA-PEFF algorithm [11]. AF likely reflects the atrial myopathy that accompanies the increase in LV filling pressures. HFpEF patients with AF may also present with functional atrial mitral or tricuspid regurgitation, associated with markedly enlarged atria and high mortality. Obesity contributes to HFpEF through many partially understood pathophysiological mechanisms in which epicardial adipose tissue could play a role [12]. Typical findings in patients with obesity include an increase in pulmonary vascular resistance that may provoke right ventricular dysfunction. These patients usually present with worse signs of peripheral and pulmonary congestion, worse functional class, and poorer quality of life [13]. Approximately 14% of patients with HFpEF also have COPD. However, the similarity in symptoms between these two conditions can lead to misdiagnosis. Patients with HFpEF and COPD are usually males and smokers and often have been prescribed diuretic therapy. They commonly exhibit significant LV concentric hypertrophy and experience a higher rate of hospitalizations and mortality [14].

Although the prevalence varies, type 2 diabetes mellitus has been identified as a contributing factor in the development of HFpEF through numerous pathophysiological mechanisms that are not yet fully understood. These mechanisms ultimately lead to concentric LV remodeling and diastolic dysfunction [3]. Patients with diabetes are at a higher risk of HF hospitalization and worse prognosis. Finally, microvascular dysfunction and obstructive CAD are highly prevalent in the HFpEF population. Patients with HFpEF and CAD are typically old women with cardiovascular risk factors (hypertension, diabetes, smoking, and chronic inflammatory diseases). Microvascular dysfunction and CAD lead to myocardial fibrosis, eventually leading to a worsening LVEF and a higher morbidity and mortality burden [15].

## 3. Clinical Characteristics and Prognosis of Patients with Chronic Kidney Disease and HFpEF

As previously discussed, patients with HFpEF and CKD have a poor quality of life and share common cardiovascular risk factors, such as arterial hypertension, diabetes, and obesity. Their prevalence increases with age [16]. Physiologically, the total glomerular filtration rate (eGFR) declines over time because of the loss of functioning nephrons. On average, this decline is estimated to be around 0.6–1 mL/min/1.73 m^2^ per year. Of note, HF is independently associated with a more pronounced decline in the eGFR after correction for risk factors associated with CKD progression, reaching up to >5 mL/min/1.73 m^2^ per year [17].

Phenotyping patients with renal dysfunction remains a real challenge; the pathophysiological mechanisms and the prognostic role of renal dysfunction may differ across the HF spectrum. CKD is in general related to more severe HF conditions and stages independently of LVEF. The relationships between CKD, older age, female sex, diabetes, and HF stage are similar in HFpEF, mildly reduced ejection fraction (HFmrEF), and HFrEF, but several studies demonstrated that CKD is more prevalent in HFpEF than in HFmrEF and HFrEF [18,19]. Other studies show a higher prevalence of CKD in patients with HFrEF [20,21]. Renal impairment could be considered a major comorbidity in HFpEF, with a significant prognostic impact without any relation with a worse HF status; conversely, in patients with HFrEF, kidney dysfunction may reflect the progression of HF, perhaps because of low cardiac output, hemodynamic hypoperfusion, and sympathetic and neurohormonal activation [22].

Renal dysfunction confers a clinically significant risk for excess mortality and HF hospitalization in all HF phenotypes; however, the literature on mortality in HFpEF and CKD shows conflicting results. In the larger meta-analyses, which included a cohort of patients with HFpEF, CKD has been found to be a more powerful predictor of death [23]. Conversely, a meta-analysis of the Global Group in Chronic Heart Failure (MAGGIC) shows a lower mortality rate and a lower association between CKD and death in patients with HFpEF than in those with HFrEF [24]. This result has been confirmed in the Swedish Heart Failure registry, in which the association between CKD and mortality is less evident in patients with HFpEF [21].

In patients with acute heart failure (AHF), we can discern between two distinct phenotypes as follows: patients with CKD at baseline and patients developing worsening renal function (WRF) during hospitalization [25]. WRF may be defined according to the time frame resolution or to its persistence. The first clinical scenario is represented by patients with good renal function and the occurrence of a “pseudo” WRF during hospitalization for AHF, usually considered secondary to decongestion therapy [26]. The increase in in-hospital creatinine usually does not persist after discharge and has no prognostic implication if the patient is well-decongested before discharge. The second scenario is represented by patients with true WRF due to congestion (increased renal venous pressure) and hypoperfusion (reduced arterial perfusion), in which renal deterioration persists, with an increase in creatinine also in the post-discharge period and with a higher burden of HF-related re-hospitalizations [27]. Finally, in the third scenario, WRF occurs in the presence of CKD related to reduced cortical blood flow and chronic glomerulosclerosis with reduced cortical wall. This phenotype is common in older patients with several comorbidities, where WRF reflects the real deterioration of renal function, with worse prognostic implications.

## 4. Pathophysiology of Chronic Kidney Disease in HFpEF

HFpEF and CKD share common pathophysiological pathways, such as systemic inflammation, oxidative stress, elevated neurohormones, venous congestion, and uncontrolled RAAS activity, all of which promote the development and perpetuation of HFpEF and renal impairment. Elevated filling pressures and decreased systolic filling are the major hemodynamic features of inadequate stroke volume, ultimately resulting in decreased cardiac output and, consequently, in renal blood flow. Water and sodium retention is the first compensatory mechanism in response to a diminished cardiac output, ultimately causing subclinical congestion and leading to a further deterioration of kidney function. Patients with HFpEF and CKD tend to present with different degrees of hypervolemia, and it might be challenging to distinguish volume overload resulting from HF or caused by progressively declining kidney function. Indeed, parameters of elevated ventricular filling pressures, such as natriuretic peptides (NPs) might also be elevated because of reduced kidney excretion in the course of CKD [28]. In patients with HF, increased abdominal pressure, or increased central venous pressure, has been associated with worsening renal function. This clinical condition is characterized by renal congestion, hypoperfusion, and increased right atrial pressure as typical manifestations. The increase in central venous pressures results in increased renal interstitial pressures that compress the tubules, intrarenal veins, and glomeruli in the encapsulated kidney leading to the “Renal Tamponade Hypothesis” [29].

In a chronic situation, the triggers and factors are involved in a complex interplay among inflammation, endothelial dysfunction, and fibrosis, eventually leading to myocardial remodeling and progressive dysfunction [30]. Recently, the key roles of systemic inflammation and the activation of profibrotic pathways have been increasingly recognized as a crucial pathological mechanism primarily leading to CKD, as well, so that CKD itself might lead to the development of HFpEF [31]. Inflammation promotes the endothelial production of reactive oxygen species. This reduces nitric oxygen (NO) availability and increases peroxynitrite, resulting in diminished cyclic guanosine monophosphate (cGMP) production by soluble guanylate cyclase and lower protein kinase G (PKG) activity [30]. Besides the above-mentioned mechanisms, fibrosis is also enhanced by the overproduction of additional peptides, such as Ang 1-7, as well as by direct proinflammatory-induced pathways involving higher circulating levels of interleukin-6 and tumor necrosis factor [32,33]. Fibroblast Growth Factor-23 (FGF-23) is increased in patients with CKD, and it has been shown to alter renal flow-mediated vascular regulation and arterial stiffness. An in vitro study on human atrial myocardiocytes demonstrated a cardiac profibrotic role and induction of myocardial hypertrophy [34]. Deficiency of Klotho protein-positive myocardial regions, the co-receptor of FGFR1, demonstrated in myocardial autoptic samples of dialysis patients with high FGF-23 serum levels, has been associated with higher cardiac fibrosis and ventricular hypertrophy with an inversely proportional relation [35]. Reduced NO levels promote impaired endothelial function which, at a coronary level, translates into increased coronary stiffness and impaired coronary blood flow. This represents the pathophysiological determinant of microcirculatory dysfunction, which is one of the most important mechanisms underlying HFpEF and CKD [30,36].

## 5. Assessment of Renal Function: Laboratory Biomarkers of Glomerular and Tubular Renal Function

The ability of serum creatinine to estimate the glomerular filtration rate is often not accurate, as it is known to be influenced by many factors as muscle mass, dietary intake, physical activity, age, and sex, independently of renal function [37,38]. Furthermore, there is a curvilinear relationship between serum creatinine and eGFR, so an increase in serum creatinine is detected only when approximately 40% to 50% of renal parenchyma is reversibly impaired or irreversibly damaged [39]. This condition could lead to a delay in the diagnosis of the early stages of acute or CKD.

Because of this tubular secreted component, the eGFR measured by 24 h creatinine clearance can exceed the eGFR derived by the reference inulin clearance by 10–40% [40]. The current criteria for the definition of CKD are based on signs of kidney damage determined by an elevated albumin or protein-to-creatinine ratio or kidney dysfunction determined by an eGFR < 60 mL/min per 1.73 m^2^ [41]. Several types of data have demonstrated that an eGFR < 60 mL/min per 1.73 m^2^ is independently associated with adverse outcomes and cardiovascular events [42]. However, this threshold does not differentiate between kidney disease related to the aging kidney, in which the GFR decreases physiologically without kidney damage, and implementation of compensation mechanisms such as an increase in the single-nephron eGFR [43]. Indeed, a subgroup analysis based on age and mortality risk in patients older than 65 years old was futile until the eGFR dropped down to 45 mL/min per 1.73 m^2^ [44]. For this reason, many experts suggest a new definition of CKD based on age-adapted eGFR.

Cystatin C, in comparison with creatinine, is freely filtered by the kidney and almost completely reabsorbed in the proximal tube; therefore, it is less influenced by patient factors such as age, gender, and skeletal muscle composition [45]. Cystatin C quantification is indicated in patients with a creatinine eGFR between 45 and 50 mL/kg per 1.73 m^2^ without other markers of kidney dysfunction and damage to confirm the diagnosis of chronic kidney disease [41]. Clinical data have shown that the determination of the eGFR using cystatin C is more accurate, particularly in patients over 70 years of age [46].

Albuminuria plays a relevant role in predicting the risk of CKD progression [42]. Normally, the glomerular capillary membrane restricts the passage of large serum proteins. The mean value of the albumin excretion rate, which begins to indicate pathological changes, is typically above 30 mg/day. This level serves as a threshold for detecting early signs of kidney damage or dysfunction, such as in the context of diabetic nephropathy or other renal diseases [47]. These alterations are exacerbated in patients with HF because of the presence of further pathological mechanisms such as endothelial dysfunction, inflammation, podocyte damage, and higher intraglomerular pressure [48]. Currently, measuring the albumin-to-creatinine ratio is preferred for estimating albuminuria because of the variability in total urinary protein components, making standardization difficult. This ratio is favored over creatinine concentration alone to avoid errors related to urine dilution or concentration, providing a more accurate assessment of renal function and early signs of kidney damage [49].

Numerous molecules present evidence as markers of renal function, although they are not yet used in clinical practice. Growth differentiating factor-15 (GDF-15) upregulates anti-inflammatory factors, such as the Klotho protein, and downregulates pro-inflammatory factors. A similar correlation between the urinary GDF-15-to-creatinine ratio to that of the urinary albumin-to-creatinine ratio and the severity of diabetic CKD was observed, together with a prognostic role in these patients (mortality and need to replace renal therapy) [50,51]. The Klotho protein mechanism of action is also associated with FGF-23, which, in a small HFpEF cohort, was shown to correlate independently with 6 MWT (*p* = 0.012) and cardiovascular events (HR 1.665; 95% CI, 1.284–2.160; *p* < 0.0001) [52]. Soluble suppression of tumorigenicity 2 (sST2) was added in the 2017 update of the ACC/AHA/HFSA 2013 HF guidelines as a biomarker of myocardial injury and fibrosis for risk stratification [53]. The diagnostic value of sST2 is less affected by decreased renal function. The combined model of sST2 and NT-proBNP for the diagnostic accuracy of HF was superior to the model of sST2 or NT-proBNP alone also in patients with CKD [54]. Furthermore, sST2 was independently associated with creatin and the urinary protein-to-creatinine ratio in a large cohort of CKD patients, as well as with renal dysfunction progression and outcome [55].

Tubular atrophy and interstitial fibrosis are the major processes associated with tubular damage. Tubular damage is a strong predictor of CKD progression, and it is associated with an increased risk of cardiovascular disease [56]. However, creatinine and the eGFR are not indicative of the healthy status of the renal tubule. Human neutrophil gelatinase-associated lipocalin (NGAL) is a glycoprotein mainly secreted by neutrophils but also released by kidney tubular cells during damage and inflammation [57,58]. Both urinary and systemic forms seem to have similar specificity and sensitivity to assess kidney damage. In particular, some studies have shown that urinary NGAL, as a marker of renal damage, anticipates detectable changes in the eGFR [59]. The systemic form of NGAL has also been shown to play a prognostic role in chronic kidney disease. Particularly in older women, NGAL values above the normal range are associated with an increased risk of renal dysfunction at 10 years [60]. In HF patients, baseline elevated values of systemic NGAL are associated with an increased risk of mortality at two years of follow-up [61]. The major limitation of the use of NGAL as an accurate marker of renal damage is linked to its overexpression in many pathologies such as cancer, neurodegenerative disease, and atherosclerosis.

N-acetyl-β-glycosaminidase (NAG) is a lysosomal enzyme that is expressed in proximal tubular cells and increasingly excreted as a potential indicator of renal tubular dysfunction. However, the results of studies focused on the role of NAG as a marker of renal dysfunction are conflicting. A case-control study showed that urinary NAG concentrations at baseline are successful in predicting micro- and macroalbuminuria in patients with type I diabetes mellitus [62]. On the other hand, NAG and other markers of tubular damage have not been shown to enhance the predictive accuracy of the baseline clinical prediction model for CKD progression based on the eGFR and urine albumin-to-creatinine ratio to assess the risk of CKD progression [63].

## 6. Therapeutic Evidence and Limitations in Patients with HFpEF and Chronic Kidney Disease

### 6.1. SGLT-2 Inhibitors

SGLT2 inhibitors (SGLT2i) have reduced cardiovascular mortality and hospitalizations due to HF in patients with HFpEF, independently from the presence of diabetes. As such, they have been recommended as a class IA drug in the treatment of HFpEF in the latest European Society of Cardiology (ESC) guidelines [64,65,66]. SGLT2i demonstrates a favorable neurohormonal, electrolyte, and safety profile in patients with both HFpEF and CKD [67,68,69]. Retrospective data suggest a nephroprotective effect even in HFpEF, with a significant decrease in the risk of end-stage renal disease [70,71,72,73]. Sotagliflozin showed a significant reduction in total HF hospitalization and CV in patients with HFpEF. However, these results should be viewed with caution because the study was underpowered for patients with HFpEF and included only patients with diabetes [74]. In the EMPEROR-Preserved trial, almost 50% of patients had a baseline eGFR <60 mL/min/1.73 m^2^ in both the empagliflozin and placebo groups. The rate of decline in the eGFR was slower in the empagliflozin group than in the placebo group (–1.25 vs. –2.62 mL/min/1.73 m^2^ per year; *p* < 0.001). Despite that, there was no significative difference in the composite renal outcome showing, a significative discordance between the effect of empagliflozin on HF outcomes and major renal events. Furthermore, the neutral effect of empagliflozin on kidney outcomes was similar across the prespecified ejection fraction subgroups of 41–49%, 50–59%, and ≥60% [75,76]. The EMPA-KIDNEY Trial investigated the effect of empagliflozin in a broad range of patients with CKD. Progression of kidney disease or death from cardiovascular causes occurred more frequently in the placebo group vs. the empagliflozin group (16.9% vs. 13.1%, HR 0.72; 0.64 to 0.82; *p* < 0.001) independently from diabetes and across subgroups of eGFR ranges. The rate of total hospitalization was significantly lower in the empagliflozin group than in the placebo group (hazard ratio, 0.86; 0.78 to 0.95; *p* = 0.003); however, there were no significant differences in terms of the composite outcome of hospitalization for HF or death from cardiovascular causes or death from any cause (in 4.5% and 5.1%, respectively) [77].

Upon the reanalysis of data using the definitions of major renal outcomes from the DAPA-HF and EMPEROR-reduced trials (defined as a ≥50% sustained decline in the eGFR and renal death), the impact of empagliflozin on major renal outcomes in the overall population was found to be neutral (HR 0.78; [+0.54, +1.13]) [70,78,79,80]. Packer et al. highlight limitations in using the eGFR slope as a surrogate for kidney disease progression in predicting drug effects on renal outcomes, particularly in HF patients, yet overall, the analysis underscores the drug’s notable safety profile for renal events [81]. The DELIVER trial confirmed these safety results for renal outcomes, with an incidence of discontinuation due to acute kidney injury (AKI) or any serious renal adverse events of 1.5% and 0.3% in the dapagliflozin group and 1.6% and 0.2% in the placebo group [65]. In a post hoc analysis, an initial and modest decline in the eGFR with dapagliflozin was observed from baseline to 1 month of follow-up, both in patients with and without recent HF hospitalization (−1.0 mL/min/1.73 mq; [−2.4, +0.4] and −4.0 mL/min/1.73 mq; [−4.3, −3.6]). Nevertheless, this decline in the eGFR did not correlate with an elevated risk of acute kidney injury (AKI) in the SGLT2i group (6.8% in the dapagliflozin group vs. 7.2% in the placebo group, *p* = 0.54). Treatment with dapagliflozin resulted in a similar attenuation of the eGFR decline from month 1 to 24 months of follow-up, independent of any previous HF hospitalization (*p* for interaction = 0.57) [82]. Post hoc analysis of the DELIVER trial showed a lower decrease in the eGFR decline rate in patients treated with dapagliflozin, despite a modest reduction following the first month of treatment (eGFR decline of 0 mL/min/1.73 m^2^ per year [−0.2, +0.3] in dapagliflozin group vs. −1.4 mL/min/1.73 m^2^ per year [−1.7, −1.1] in the placebo group, *p* < 0.001). This renal protective effect was present independently of the eGFR at baseline. Furthermore, in patients with an LVEF range of 50–59%, the efficacy in reducing the eGFR reduction rate was similar to that in the range with LVEF greater than 60% [83].

These findings indicate that SGLT2 inhibitors decelerate the long-term decline in kidney function without elevating the risk of serious renal adverse events, thereby offering sustained cardiovascular and renal protective effects over time [84] (Table 1).

### 6.2. Mineral Receptor Antagonist

TOPCAT was a double-blind phase III clinical trial on spironolactone in patients with HF and LVEF ≥ 45%. The composite endpoint did not occur with statistically significant differences between the treatment and placebo arms [85]. However, a successive sub-analysis found inconsistency due to marked heterogeneity in the Eastern Europe cohort and the American cohort. Further analysis considering only the American cohort showed greater efficacy of spironolactone in preventing the endpoint outcomes (i.e., cardiovascular death, hospitalization, and resuscitated arrest) in patients with an eGFR > 60 mL/min/1.73 mq (HR 0.91 [+0.73, +1.14] vs. HR 0.74; [+0.57, +0.97]). Even considering the median eGFR, the drug was more effective in patients with values higher than the median, suggesting a greater efficacy of MRAs in preventing cardiovascular death, hospitalization, and resuscitated arrest in patients without CKD [86].

Additionally, in another sub-analysis of the TOPCAT trial, visit-to-visit variability in kidney function, as measured by blood urea nitrogen (BUN) and serum creatinine levels, was found to be associated with poorer clinical cardiovascular outcomes in both the spironolactone and placebo arms [87]. These data suggest that MRAs are more effective in preventing cardiovascular adverse events in patients with HFpEF with preserved renal function compared with patients with a reduced eGFR < 60 mL/min/1.73 mq.

Finerenone is a third-generation MRA with no significant interaction with the estrogen receptor, which has a lower incidence rate of hyperkalemia and kidney injury. The sub-analysis of the FIDELIO-DKD trial, stratifying patients based on a history of heart failure with a left ventricular ejection fraction (LVEF) ≥40% or not, revealed a trend toward a reduction in time-to-event cardiovascular outcomes in the finerenone arm, although this trend did not reach statistical significance [88]. Further ongoing studies on finerenone include the FINEARTS-HF and the MIRACLE trials. The FINEARTS-HF trial is a phase II study currently investigating the benefit of finerenone on cardiovascular death and HF in patients with HFpEF with or without type 2 diabetes, whereas the MIRACLE trial is a phase II study investigating the MR modulator AZD9977 on top of the SGLT2i dapaglifozin in patients with HF with LVEF ≥ 40% and CKD. The sub-analysis of the FIDELIO-DKD trial dividing patients according to history of HF with LVEF ≥ 40% or not, has evidenced a trend toward a reduction in the time-to-event cardiovascular outcomes without statistical significance in the finerenone arm [88].

### 6.3. Sacubitril/Valsartan

Pooled data derived from the PARADIGM-HF and PARAGON-HF trials showed a lower rate of renal adverse outcome (defined as a decrease in the eGFR of ≥50 mL/min/1.73 mq, end-stage renal disease, and renal death) in patients treated with sacubitril/valsartan. However, sacubitril/valsartan was beneficial only in patients with LVEF <60%. In the subgroup analysis based on an eGFR below or above 50 mL/min/1.73 mq, the decreased number of events led to the loss of statistical significance. Nonetheless, a trend indicating a greater reduction in total worsening HF events and cardiovascular deaths was observed in the eGFR ≤ 60 mL/min/1.73 m^2^ subgroup [89]. In the PARAGON-HF trial, there was no observed benefit in reducing heart failure hospitalizations and cardiovascular deaths across the entire study population. However, in a subgroup analysis, patients with an eGFR of less than 60 mL/min/1.73 m^2^ demonstrated a reduction in the prespecified primary endpoint (HR 0.79, 95% CI 0.66–0.95). In the sacubitril/valsartan arm, a preventive effect was observed with a lower incidence of worsening renal function [90]. Combining sacubitril/valsartan with MRA did not demonstrate a further reduction in renal outcomes compared to sacubitril/valsartan alone. However, the combination therapy still exhibited a lower rate of eGFR decline [89].

In the PARAMOUNT study, a phase II trial showcasing a reduction in N-terminal pro-B-type natriuretic peptide (NT-proBNP) after 3 months in patients with HFpEF treated with sacubitril/valsartan at maximum dose (200 mg), there was no statistically significant difference between patients with eGFR < 60 mL/min/1.73 mq and patients with a eGFR > 60 mL/min/1.73 mq. Moreover, a relatively short follow-up of 3 months proved to be adequate to demonstrate a lesser decrease in renal function between the sacubitril/valsartan and valsartan treatment groups (−1.6 vs. −5.2 mL/min/1.73 m^2^, *p* = 0.007). However, this result was partially offset by a greater increase in the sacubitril/valsartan treatment group in the albumin-to-creatinine ratio (from 1.9 to 2.9 mg/mmol vs. from 2.0 to 2.0 mg/mmol) [91].

## 7. Angiotensin-Converting Enzyme Inhibitors/Angiotensin II Receptor Blockers

RAAS inhibitors have not been shown to reduce mortality or morbidity in patients with HFpEF. The CHARM-PRESERVED trial, conducted in patients with LVEF ≥ 40%, showed a trend in favor of candesartan for a reduction in new hospitalization for HF in patients with HFpEF [92]. In a retrospective analysis, a greater incidence of worsening renal failure (WRF) was found in the candesartan group (OR 2.44 [+1.88, +3.19], *p* < 0.001) compared with the placebo. Patients with WRF more frequently had an ischemic HFpEF phenotype; however, the onset of WRF was not associated with an increased risk of HF hospitalization, although a positive trend was noted [93].

In the PEP-HF trial, including HF patients without systolic dysfunction randomized to perindopril or placebo, no significant differences in serum creatinine at 1 year were observed [94]. In the I-PRESERVE trial, irbesartan failed to improve cardiovascular outcomes in patients with HFpEF. Significantly, a doubling of the serum creatinine level was observed in at least one measurement in 6% of patients in the irbesartan group and in 4% of patients in the placebo group (*p* < 0.001), with no difference in the rates of serious adverse events attributable to renal dysfunction between the two groups (3% vs. 3%; *p* = 0.29) [95]. A post hoc analysis showed that WRF occurred more frequently in the irbesartan group, which was associated with an increased risk for the primary outcome. The mean overall decrease in the eGFR at 30 months was –3.4 mL/min/1.73 m^2^ in the placebo group compared with –7.2 mL/min/1.73 m^2^ in the irbesartan group. However, the interaction between treatment and WRF on outcome was not significant in an adjusted analysis, except for all-cause mortality [93].

RAAS antagonists affect renal hemodynamics, resulting in increased creatinine levels. Importantly, this kind of creatinine increase is generally benign and not related to adverse prognosis [28]. Because of their renal protective effects and positive prognostic effects in the elderly, angiotensin-converting enzyme (ACE) inhibitors are often administered as one of the first-line therapies to treat arterial hypertension [96]. Accordingly, the SOLVD trial (Studies of Left Ventricular Dysfunction) revealed that early WRF following the initiation of ACE inhibitors did not lead to a higher mortality risk. Indeed, in patients who continued the therapy despite the formal renal function decrease, a benefit in terms of mortality persisted despite the creatinine increase [97]. In the SPRINT (Systolic Blood Pressure Intervention Trial) trial, including up to 28% of hypertensive patients also presenting with CKD, intensive treatment against arterial hypertension significantly reduced the primary endpoint (a composite of myocardial infarction, stroke, HF, or cardiovascular death) [98]. Importantly, no evidence of a differential treatment effect on cardiovascular outcomes between patients with or without CKD was found. In a retrospective analysis of the DOSE study (Diuretic Optimization Strategies Evaluation) including patients with HF, deterioration in renal function did not correlate with an increased risk for the combined endpoint. However, improved renal function was linked to a higher risk of death and hospitalization. This might be explained by the fact that patients were more severely ill or inadequately decongested, as only ca. 8% were deemed congestion-free by physicians. Although more patients (19%) with WRF achieved congestion-free status at 72 h, the onset of WRF was associated with a significantly shorter duration of diuretic treatment, potentially impacting readmission because of incomplete decongestion [99].

There are no specific data regarding the treatment in which ACE inhibitors reduce the slope of the eGFR decline compared with placebos. However, the benefits in patients with HF and hypertensive heart disease treated with these drugs are sustained despite an initial drop in the eGFR. Finally, ACE inhibitors showed their reno-protective effects in patients with CKD and diabetes. Fewer data are available for angiotensin-receptor blockers (ARBs), but a single study on HFpEF and CKD showed that treatment with ARBs was associated with a modest reduction in all-cause mortality in older patients including those with more advanced CKD [100].

## 8. Beta-Blockers

Beta-blocker therapy has been demonstrated to be generally safe regarding renal function in patients with CKD, although it is not completely neutral, as shown by the meta-analysis of the COPERNICUS and CAPRICORN trials [101]. This pharmacological class is reasonably useful in the hypertensive phenotype of HFpEF, even though in a large real-world cohort of CKD patients from the Sweden National Registry, beta-blockers did not show a significant preventive effect with respect to all-cause and cardiac mortality [102]. In particular, beta-blockers have a deleterious effect on patients with HFpEF presenting with inadequate chronotropism. In a sub-analysis of the SENIORS trial, in which patients were grouped according to LVEF, the beta-blocker effect regarding the primary endpoint (cardiovascular death and hospitalization for decompensation) was neutral for patients with both HFpEF and HFrEF [103]. No interaction was observed between the effects of nebivolol on primary outcomes and renal function (*p* = 0.442) [104].

## 9. Hyperkalemia in HF and CKD

Hyperkalemia, defined as a serum potassium >5 mmol/L, is associated with adverse outcomes and affects clinical presentation and management [105]. It can be classified as mild (>5.0 to <5.5 mmol/L), moderate (5.5 to 6.0 mmol/L), or severe (>6.0 mmol/L) [106]. Potassium homeostasis is ensured by the kidneys, which handle 90–95% of its excretion, while the colon is responsible for the remaining portion [107,108]. Normally, potassium ingested through the diet is readily excreted by the kidney. However, in the case of renal insufficiency, its elimination is reduced. In uremic rats, a decrease in mRNA transcription of the alpha-1 isoform of Na-K-ATPase caused a reduced activity of the Na-K pump with impaired intracellular uptake. In addition, metabolic acidosis promotes an extracellular shift in K+ that is exchanged with protons. Finally, in advanced kidney disease, the onset of an oliguric state reduces the nephrons’ ability to eliminate potassium [109,110,111]. In patients with HF, both comorbidities and medications can increase the risk of hyperkalemia. Hyperglycemia can lead to the passage of water and K+ from the intracellular to the extracellular compartment, while a low insulin concentration reduces the activity of the Na-K pump [112]. Non-cardioselective beta-blockers could lead to an extracellular K+ shift through direct renin inhibition [113]. Despite the reduction in aldosterone levels, hyperkalemia risk is generally low following a single RAAS blockade therapy, but it is higher in patients with CKD, especially in the case of concurrent HF, diabetes, or MRA use [114] MRA therapy is often discontinued in patients with an eGFR < 30 mL/min because of a fear of a relevant potassium increase, but recent data from a Swedish registry revealed that their safety profile is persistent across the whole eGFR spectrum [115]. ARNIs are a safe option in CKD patients: indeed, sacubitril/valsartan demonstrated a lower risk of severe hyperkalemia in patients treated with MRAs compared with enalapril [116]. CKD and hyperkalemia risk remain the most important factors for the underuse and discontinuation of RAASi drugs, thus leading to an increased risk of adverse outcomes [117,118]. As such, in the case of mild hyperkalemia, the continuation of these medications is recommended [119]. The presence of K+ levels >6 mmol/L and “de novo ECG alterations” represents a life-threatening situation requiring hospitalization: in this case, calcium gluconate can reduce the cellular excitability threshold and thus the risk of life-threatening arrhythmias, while correction of metabolic acidosis and drugs such as insulin or salbutamol can be used acutely to lower potassium levels. Management of moderate or severe K+ levels, in the absence of acute emergencies (e.g., arrhythmias or AKI requiring hemodialysis), can rely on various therapeutic options. In general, K^+^ >6.5 mmol/L requires discontinuation of drugs acting on the RAAS [120]. In addition, the use of loop diuretics and thiazides lowers potassium levels by increasing its urinary excretion, although they must be used with caution in patients with severe kidney disease [121]. SGLT2i shows a reduction in hyperkalemia risk in patients with CKD and diabetes [122]. When K^+^ persists at >5.5 mmol/L despite these measures, a 50% reduction in or a short-term suspension of RAASi and MRAs should be considered, followed by close monitoring of the potassium levels and possibly a reintroduction of these medications as soon as possible [66]. A novel alternative approach is the utilization of K+ binders. These drugs are cation exchangers acting in the gastrointestinal tract. Sodium polystyrene sulfonate, which exchanges sodium for potassium, has been available for several years, but its prescription is limited by the risk of severe gastrointestinal events [123]. Recently, patiromer and sodium zirconium cyclosilicate (SZC) were approved for hyperkalemia management, ameliorating the management of RAASi therapy and diet in CKD patients. Patiromer exchanges potassium for calcium: in a phase 2 study, patients affected by diabetes mellitus and hypertension receiving patiromer increased their adherence to RAASi therapies, with up to 100% maintaining appropriate potassium levels. These data were confirmed in a post hoc analysis of older patients with diabetic CKD [124,125]. Furthermore, in a dedicated trial for patients with RAASi-related hyperkalemia, patiromer was associated with significantly lower serum potassium levels, fewer hyperkalemia episodes, concurrent use of higher doses of MRAs, and overall higher RAASi administration [126]. SZC selectively captures potassium in exchange for hydrogen and sodium ions in the gastrointestinal lumen; it reduces serum potassium to normal levels within 48 h with prolonged effects over time, even in patients with CKD and RAASi [127]. Furthermore, SZC efficacy has been proven in patients on hemodialysis [128]. Both drugs are well tolerated: constipation and electrolyte disturbances are the primary reported side effects for both drugs, whereas SZC may lead to a higher incidence of edema.

## 10. Potential Strategy for the Correct Use of Heart Failure Treatments According to Renal Function

A comprehensive characterization of HFpEF profiles could facilitate the targeted application of both established and novel HF drugs. Indeed, sub-analyses from previous trials have indicated advantages in specific populations, such as in patients with specific cutoffs of LVEF or the role of gender, as demonstrated in TOPCAT or PARAGON-HF trials [90,129]. At the same time, improved HFpEF profiling could pave the way for new therapies, as individualized indications have the potential to translate into therapeutic success. This has been the case for the GLP1 receptor antagonist semaglutide, which showed symptom and functional improvement in patients with HFpEF and obesity [130]. New studies are investigating the correlation between distinct comorbidity profiles and candidate genes, aiming to improve the diagnosis and identify treatment targets for HFpEF [4].

In this context, CKD represents a real “nightmare” when starting and optimizing HF therapy. Additional studies are recommended to improve our knowledge of medication in patients with HFpEF and CKD. As such, patients with severe renal impairment should be included in future trials. Actually, SGLT-2i is recommended as the first-line treatment in all patients with HFpEF and presents a safe profile in patients with CKD. The use of RAAS inhibitors in the HFpEF phenotype with hypertensive heart disease is recommended in all patients with mild to moderate CKD. The prescription of an MRA antagonist may be considered in patients with HFpEF after a careful evaluation of hyperkalemia.

**Figure 1:**
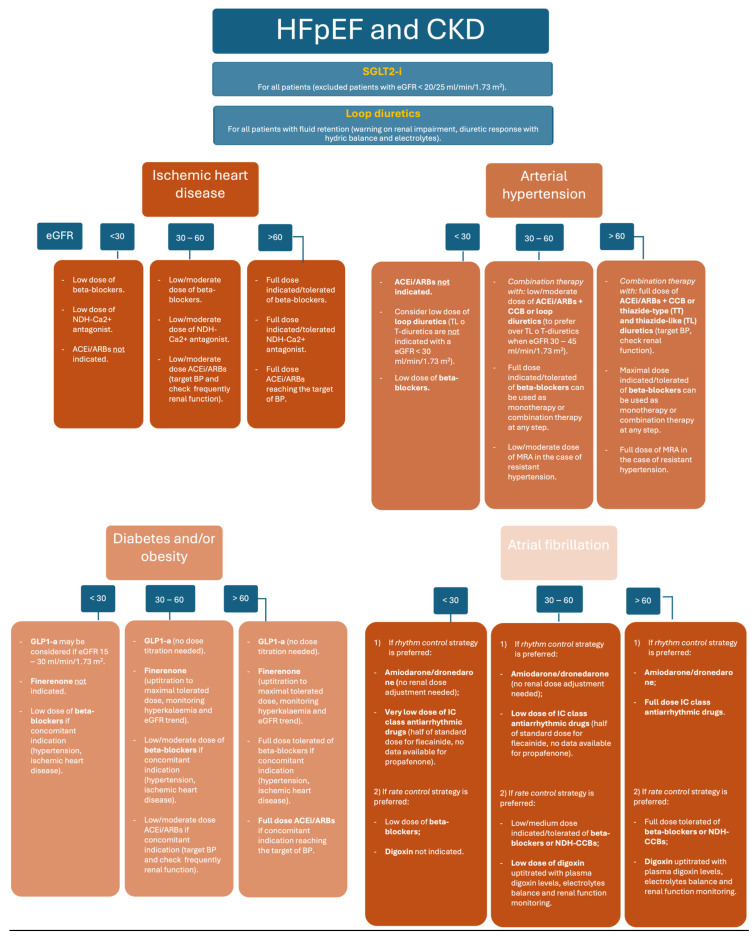
Management of HF drugs in HFpEF and different phenotypes. HFpEF (heart failure with preserved ejection fraction), CKD (chronic kidney disease), SGLT2-i (sodium–glucose transport protein 2 inhibitors), eGFR (estimated glomerular filtration rate), NDH-Ca^2+^ antagonists (non-dihydropyridine calcium channel antagonists), ACEi (angiotensin-converting enzyme inhibitors), ARBs (angiotensin II receptor blockers), CCB (calcium channel blockers), T-diuretics (thiazide diuretics), TL-diuretics (thiazide-like diuretic), BP (blood pressure), MRA (mineral receptor antagonist), GLP-1a (glucagon-like peptide 1 agonists).

The combination of multiple drugs may suddenly change renal physiology, thus leading to an acute decline in the eGFR, even in patients with a previous normal renal function (Figure 1). Usually, the initial mild dip in the eGFR (20–30% of eGFR reduction from baseline) is reversible, and renal function returns to baseline levels during the follow-up. In patients with severe renal dysfunction, the combination of multiple drugs may become deleterious and should be avoided. A careful monitoring of the renal function and electrolytes through blood samples should be performed 3 or 4 weeks after the beginning of HF therapy in order to avoid sudden eGFR deterioration and K^+^ increase. Overall, we recommend checking renal function and K^+^ every 2 or 3 months, during up-titration of HF therapy up to the maximally tolerated dose, in patients with renal dysfunction. Whenever serum creatinine increases by >50% or above 3.5 mg/dL, treatment should be discontinued [131]. The down-titration of HF drugs is recommended in the presence of K^+^ levels between 5.5 mmol/L and 6 mmol/L, whereas temporary discontinuation is advised for potassium levels >6 mmol/L. In patients with normal renal function and isolated K^+^ increase, novel K^+^ binders such as patiromer and sodium zirconium cyclosilicate substantially allow for reduced reversibly serum K^+^ levels in the long-term, thus allowing the up-titration and maintenance of HF treatment.

In AF and HFpEF phenotypes, there is no requirement for dosage adjustments or supplemental dosing of amiodarone in patients with renal impairment or in dialysis. On the contrary, a lower eGFR to 30 mL/min/1.73 m^2^ contraindicates digoxin therapy, while CKD IIIA or IIIB stages require a lower starting dose and careful titration by monitoring serum digoxin and electrolyte levels. In these patients, it could be useful to uptritate other rate-controlling agents (i.e., b-blockers, calcium antagonists) in order to avoid digoxin adverse effects. Concerning statin therapy in patients with CKD and HF, the use of rosuvastatin is associated with an increased risk of proteinuria and renal replacement therapy compared with atorvastatin. A moderate-intensity dose of atorvastatin has fewer side effects on renal function than that of rosuvastatin [132,133].

## 11. Future Perspectives

In recent years, many resources have been invested in new treatments for HFpEF. Novel devices have been tested, the most investigated being atrial shunt devices (ASD), such as Corvia ASD, which, in the REDUCE-HF LAP HF II trial, was not shown to reduce cardiovascular events or to improve the quality of life of patients [134]. FROST-HF trial on Atrial Flow Regulator in patients with HFpEF and HFrEF is still recruiting [NCT05136820]. Drugs from distinct therapeutic areas are currently being tested in different phenotypes of HFpEF. The SERENADE trial, which aims to study the effect of macitentant (endothelin A and endothelin B antagonist) in patients with cardiac remodeling and pulmonary vascular disease, is still ongoing [NCT03153111]. A phase 2a proof of concept study, EMBARK-HFPEF, aims to assess the preliminary efficacy and safety of mavacamten in patients with HFpEF and study the effects on NT-proBNP and cTnT serum levels [NCT04766892]. Inflammation has a key role in the pathophysiology of HFpEF. In the COLpEF study, an RCT of colchicine versus placebo in patients with HFpEF and HFmrEF, will measure the anti-inflammatory effect of colchicine as changes in hs-CRP serum levels and of hemodynamic stress indexes, such as NTproBNP, biomarkers of myocardial injury, LV diastolic dysfunction, and patients symptom [NCT04857931]. Considered a promising therapeutic target in the treatment of HFpEF, monoclonal antibody drugs against proinflammatory factors are currently under development.

## 12. Conclusions

HF and CKD are risk factors for each other, sharing several comorbidities and pathophysiologic pathways. When evaluating patients with HF, the unified concept of cardiorenal syndrome as a single entity should be taken into account to address symptom status, disease progression, and treatment options. Starting and up-titrating HF treatment is mandatory in patients with HF and CKD. In most cases, renal impairment after starting HF treatment is transitory, and kidney function tends to return to its previous levels or remain stable in the long term. Over the years, the false myth of administering inadequate target doses or withdrawing HF therapies to avoid end-stage renal disease resulted in lower use of these lifesaving therapies, with a significant negative impact on HF prognosis. However, the combination of multiple HF therapies requires caution and frequent monitoring of renal function and potassium levels, in particular during the initial up-titration phase.

## Figures and Tables

**Table 1:** Renal results from RCTs and post-hoc analysis on SGLT2-i.

			Renal Analysis and Endpoints			
Study (Year)	Patients and Design of the Study	Primary Endopoint	Baseline eGFR	Safety Outcome	Progression of kidney Disfunction (Based on eGFR Trend)	Efficacy Outcome
*SGLT2-i*						
EMPEROR-preserved (2021) [75]	*Randomized with empagliflozin* A total of 5988 adults (≥18 years of age), LVEF of more than 40%, and NYHA classes II, III, or IV symptoms. Median follow up of 26 months.	Composite of death from cardiovascular causes or hospitalization for HF (first or recurrent).	60.6 ± 19.8 mL/min/1.73 m^2^ in the empagliflozin group and 60.6 ± 19.9 mL/min/1.73 m^2^ in the placebo group	NA	The rate of decline in the eGFR was slower in the empagliflozin group than in the placebo group (–1.25 vs. –2.62 mL per minute per 1.73 m^2^ per year; *p* < 0.001).	No significative difference in the composite renal outcome: 3.6%, with 2.1 events per 100 patient-year in the empagliflozin group versus 3.7% and 2.2 events per 100 patient-year in the placebo group (HR 0.95, 95% CI).
DELIVER (2022) [65]	*Randomized with dapagliflozin* A total of 10,584 adults (≥18 years of age), LVEF of more than 40%, and NYHA classes II–IV, including patients with improved LVEF.	Composite of worsening HF (hospitalization or an urgent visit resulting in intravenous therapy for HF) or death from cardiovascular causes.	61 ± 19 mL/min/1.73 m^2^ in both groups	Incidence of AKI and any serious renal adverse event that led to treatment discontinuation were, respectively, 1.5% and 0.3% in the dapagliflozin group vs. 1.6% and 0.2% in the placebo group.	NA	NA
S. Chatur et al. (2023) [82]	*Pre-specified analysis* Same population as the DELIVER-trial.		61 ± 19 mL/min/1.73 m^2^ in both groups	Already analyzed in the RCT.	Initial decline in the eGFR with dapagliflozin from baseline to 1 month of follow-up of −1.0 (−2.4, +0.4) mL/min/1.73 m^2^ in patients with recent HF hospitalization, and of −4.0 (−4.3, −3.6) mL/min/1.73 m^2^ in patients without recent HF hospitalization. Similar attenuation of eGFR decline from month 1 to 24 months in patients with (+0.03 [−0.1, +0.2] mL/min/1.73 m^2^) and without (+0.08 [+0.04, +0.12] mL/min/1.73 m^2^) recent HF hospitalization (*p*-interaction = 0.57). Similar effect of dapagliflozin treatment on total slope from baseline to end of follow-up in patients with (+0.02 [−0.12, +0.16] mL/min/1.73 m^2^) and without (+0.04 [+0.01, +0.07] mL/min/1.73 m^2^) recent HF hospitalization (*p*-interaction = 0.66).	NA
F.R. Mc Causlan et al. [83]	*Post hoc analysis* Same population as the DELIVER-trial.		61 ± 19 mL/min/1.73 m^2^ in both groups	Already analyzed in the RCT.	Initial acute decline in the eGFR in the dapagliflozin group between baseline and month 1 (−3.7; 95% CI, −4.0 to −3.3 mL/min/1.73 m^2^), compared with those assigned to the placebo (−0.4; 95% CI, −0.8 to 0 mL/min/1.73 m^2^). Between month 1 and the end of the trial, the mean decline in the eGFR was 0 mL/min/1.73 m^2^ per year (95% CI, −0.2 to 0.3) for those assigned to dapagliflozin, compared with −1.4 mL/min/1.73 m^2^ per year (95% CI −1.7 to −1.1) for those assigned to the placebo, with a mean difference of 1.4 (95% CI, 1.0–1.8) mL/min/1.73 m^2^ per year (*p* < 0.001). In the LVEF 50–59% and LVEF ≥ 60% groups, the dapagliflozin arm showed a chronic eGFR slope of 0.3 (−0.1, 0.8) vs. −1.7 (−2.2, −1.2) in the placebo group (*p*-interaction < 0.001) and of 0.1 (−0.6, 0.4) in the dapagliflozin group vs. −1.6 (−2.0, −1.1) in the placebo group, (*p*-interaction < 0.001), respectively. In patients with LVEF ≤49% the difference between the two arms was less pronounced: the eGFR slope in the dapagliflozin group was −0.1 (−0.7, 0.4) vs. −0.8 (−1.3, −0.3) in the placebo group (*p*-interaction < 0.001).	The overall incidence rate of the post hoc kidney composite outcome was similar between the two groups (2.5% in the dapagliflozin group and 2.3% in the placebo group (HR, 1.08; 95% CI, 0.79–1.49)).
S. Chatur et al. (2023) [69]	*Pre-specified analysis* Same population as the DELIVER-trial.		61 ± 19 mL/min/1.73 m^2^ in both groups	Already analyzed in the RCT.	NA	Dapagliflozin reduced new initiation of loop diuretics by 32% [hazard ratio (HR) 0.68; 95% confidence interval (CI): 0.55–0.84, *p* < 0.001]. First sustained loop diuretic dose increases were less frequent, and sustained dose decreases were more frequent in patients treated with dapagliflozin: net difference of −6.5% (95% CI: −9.4 to −3.6; *p* < 0.001).

AKI (acute kidney injury), eGFR (estimated glomerular filtration rate), HF (heart failure), LVEF (left ventricular ejection fraction), NYHA (New York Heart Association), RCT (randomized controlled trial), SGLT2-I (sodium–glucose-linked transporter 2 inhibitors). Light orange underlines the results of studies, while white and light blue shows the study design.
